# Geographic Distribution of MERS Coronavirus among Dromedary Camels, Africa

**DOI:** 10.3201/eid2008.140590

**Published:** 2014-08

**Authors:** Chantal B.E.M. Reusken, Lilia Messadi, Ashenafi Feyisa, Hussaini Ularamu, Gert-Jan Godeke, Agom Danmarwa, Fufa Dawo, Mohamed Jemli, Simenew Melaku, David Shamaki, Yusuf Woma, Yiltawe Wungak, Endrias Zewdu Gebremedhin, Ilse Zutt, Berend-Jan Bosch, Bart L. Haagmans, Marion P.G. Koopmans

**Affiliations:** Netherlands Centre for Infectious Disease Control, Bilthoven, the Netherlands (C.B.E.M. Reusken, G.-J. Godeke, I. Zutt, M.P.G. Koopmans);; Erasmus Medical Center, Rotterdam, the Netherlands (C.B.E.M. Reusken, B.L. Haagmans, M.P.G. Koopmans);; National Veterinary Medicine School, University of La Manouba, Sidi Thabet, Tunisia (L. Messadi, M. Jemli);; Addis Ababa University College of Veterinary Medicine and Agriculture, Bishoftu, Ethiopia (A. Feyisa, F. Dawo, S. Melaku, E. Z. Gebremedhin);; National Veterinary Research Institute, Vom, Nigeria (H. Ularamu, A. Danmarwa, D. Shamaki, Y. Woma, Y. Wungak); Faculty of Veterinary Medicine, Utrecht University, Utrecht, the Netherlands (B.-J. Bosch); 1 These authors contributed equally to this article.

**Keywords:** Middle East respiratory syndrome, MERS, Coronaviridae, beta-coronavirus, zoonoses, pneumonia, coronavirus infections, disease reservoirs, camels, Africa, Arabian Peninsula, viruses

## Abstract

We found serologic evidence for the circulation of Middle East respiratory syndrome coronavirus among dromedary camels in Nigeria, Tunisia, and Ethiopia. Circulation of the virus among dromedaries across broad areas of Africa may indicate that this disease is currently underdiagnosed in humans outside the Arabian Peninsula.

A novel betacoronavirus, Middle East respiratory syndrome coronavirus (MERS-CoV), was identified as the cause of severe respiratory disease in humans during 2012 (*1*). In August 2013, dromedary camels (*Camelus dromedarius*) were implicated for the first time as a possible source for human infection on the basis of the presence of MERS-CoV neutralizing antibodies in dromedaries from Oman and the Canary Islands of Spain (*2*). Since then, the presence of MERS-CoV antibodies in dromedaries has been reported in Jordan (*3*), Egypt (*4*,*5*), the United Arab Emirates (*6*,*7*), and Saudi Arabia (*8*,*9*). In October 2013, analysis of an outbreak associated with 1 barn in Qatar (*10*) found dromedaries and humans to be infected with nearly identical strains of MERS-CoV. Further proof of widespread circulation of MERS-CoV among dromedaries was provided by studies from Egypt and Saudi Arabia (*5*,*9*). These findings have raised questions about the geographic distribution of MERS-CoV among camel populations elsewhere. Here, we report our assessment of the geographic distribution of MERS-CoV circulation among dromedaries in Africa by serologic investigation of convenience samples from these animals in Nigeria, Tunisia, and Ethiopia.

## The Study

**Figure 1:**
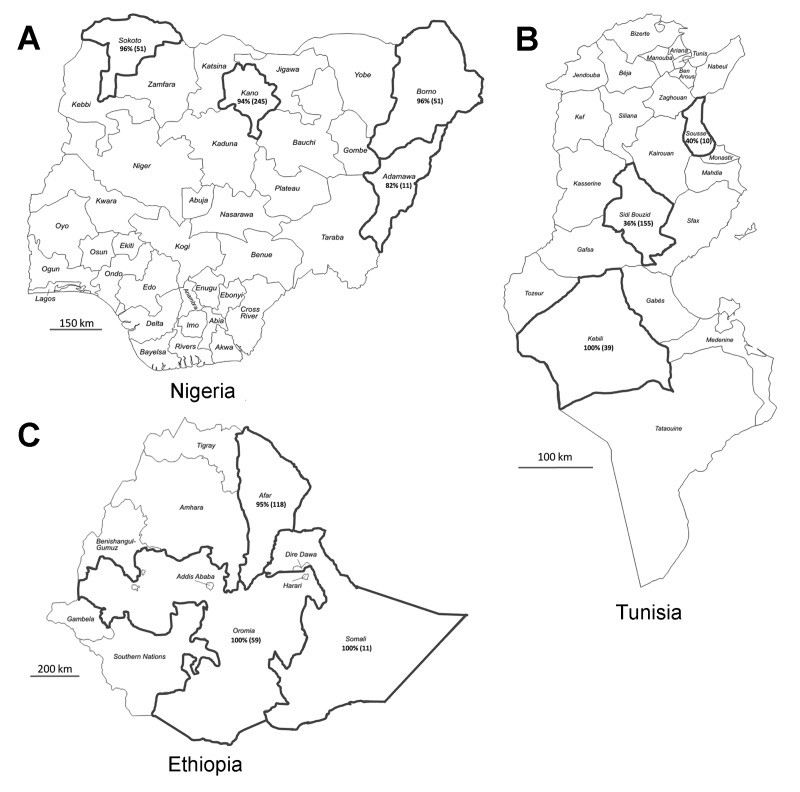
Countries and provinces sampled in this study: A) Nigeria, B)Tunisia, and C) Ethiopia. Black outline indicates provinces in which samples were collected. Serologic results are indicated in each province as percentage seropositive for Middle East respiratory syndrome coronavirus (total no. dromedaries tested). Maps adapted from http://d-maps.com/index.php

In Nigeria, serum samples from 358 dromedaries that were raised for meat production were collected at abattoirs in 4 provinces (Kano, n = 245; Sokoto, n = 51; Borno, n = 51; and Adamawa, n = 11; Figure 1, panel A) during 2010–2011 for testing for peste des petits ruminants virus. The ages of the animals ranged from 4 to 15 years. The abattoirs also served the neighboring countries of Chad, Niger, and the Central African Republic. In Tunisia, serum samples from 204 dromedaries that were 1 to 16 years of age were collected in 3 provinces in 2009 and 2013 (Figure 1, panel B). Samples were collected from 155 dromedaries in Sidi Bouzid Province from 27 herds that were kept for meat production and from 39 dromedaries in Kebili Province from 16 herds that were kept for tourist rides; samples from both provinces had originally been collected for a study investigating the presence of *Anaplasma phagocytophilum.* Samples were collected from 10 dromedaries from Sousse Province that were kept for meat production because they were suspected of being infected with *Trypanosoma evansi*. In Ethiopia, samples from 188 dromedaries, 1 to 13 years of age, were collected as part of a study evaluating the presence of toxoplasmosis and respiratory tract diseases in 3 provinces (Afar, n = 118; Somalia, n = 11; and Oromia, n = 59; Figure 1, panel C) during 2011–2013. All samples were taken by jugular vein puncture according to local laws, and serum samples were stored at −20°C until testing. All serum samples were shipped to the Erasmus MC laboratory in the Netherlands in agreement with Dutch import regulations.

The serum samples were tested for the presence of IgG antibodies reactive with S1 antigens against MERS-CoV (residues 1–747), severe acute respiratory syndrome CoV (residues 1–676), and human CoV OC43 (residues 1–760) by using extensively validated protein-microarray technology, as described (*2*,*3*,*6*,*11*). Results were expressed as relative mean fluorescent intensity (RFU) for each set of quadruplicate spots of antigen, with a cutoff of 4,000 RFU as used by Meyer et al. (*6*). Human CoV OC43 S1 was used as a proxy for bovine coronavirus (ΒCoV), the latter of which is known to circulate commonly in dromedaries (*7*,*12*). High percentages of animals seropositive for MERS-CoV were observed in Nigeria and Ethiopia; the overall seropositivity was 94% in adult dromedaries in Nigeria and 93% and 97% for juvenile and adult animals, respectively, in Ethiopia (Table 1). All provinces in which dromedaries were sampled in both countries showed high rates of seropositivity (Figure 1). The overall seropositivity in dromedaries in Tunisia was 30% for animals ≤2 years of age and 54% for adult animals. Seropositivity of 36% and 40% was observed in Sidi Bouzid and Sousse Provinces, respectively, and 100% of the dromedaries in the southern province of Kebili were seropositive. Array results were confirmed on a selection of positive and negative serum samples (n = 14 per country) in MERS-CoV neutralization tests performed as described (*2*) (Table 2). Serum samples from 72%, 82%, and 67% of the dromedaries from Nigeria, Ethiopia, and Tunisia, respectively, reacted with the OC43 antigen, confirming common circulation of ΒCoV in camelids (*7*,*12*). All samples tested negative for severe acute respiratory syndrome CoV (data not shown).

**Table 1:** Overview of serologic evidence for Middle East respiratory syndrome coronavirus among dromedary camels, Africa and the Arabian Peninsula

Country	Year	No. camels*	% Middle East respiratory syndrome coronavirus antibodies†	Reference
United Arab Emirates	2013	500 (A,J)	96‡,§,¶	(*6*)
	2013	59 (A)	97# , 100**, 98§	(*7*)
	2003	151 (A)	100§,‡	(*6*)
Egypt	2013	110 (A)	94§, 98††	(*4*)
	2013	17 (A)	82††	(*5*)
Spain (Canary Islands)	2012–2013	97 (A)	14¶,§	(*2*)
	2012–2013	8 (J)	13¶,§	(*2*)
Ethiopia	2010–2011	31 (J)	93¶	This study
	2010–2011	157 (A)	97¶	This study
Ethiopia, Sudan	2013	35 (A)	97††	(*5*)
Jordan	2013	11 (J)	100¶,§	(*3*)
Nigeria	2010–2011	358 (A)	94¶	This study
Oman	2013	50 (A)	100¶,§	(*2*)
Qatar	2013	14 (A)	100¶,§	(*10*)
Saudi Arabia	2010–2013	65 (J)	72††	(*8*)
	2010–2013	245 (A)	95††	(*8*)
	2013	104 (J)	55‡‡	(*9*)
	2013	98 (A)	95‡‡	(*9*)
	2010	21 (J)	76‡‡	(*9*)
	2010	23 (A)	91‡‡	(*9*)
	2009	56 (J)	72‡‡	(*9*)
	2009	26 (A)	92‡‡	(*9*)
	2004	6 (A)	100‡‡	(*9*)
	1996	6 (A)	100‡‡	(*9*)
	1994	123 (A)	93‡‡	(*9*)
	1993	2 (A)	100‡‡	(*9*)
	1992	1 (A)	100‡‡	(*9*)
Tunisia	2009	46 (J)	30¶	This study
	2009	158 (A)	54¶	This study

*Camel age range indicated where known: J, juvenile ≤2 y of age ; A, adult >2 y of age. †As determined by: ‡recombinant spike IFA. §Neutralization test. ¶S1 micro-array.  #Nucleocapsid Western blot.  **Whole virus IFA.  ††Pseudoparticle neutralization test. ‡‡Complete virus infected cell ELISA.

**Table 2:** Background data and Middle East respiratory syndrome coronavirus serology results of selected camel serum samples from Nigeria, Ethiopia, and Tunisia*

Country, Sample ID	Region	Age	Sex	MERS S1* (1:20)	MERS S1 (1:320)	MERS (1:640)	VNT
Nigeria							
1	Kano	7	M	63410	52,254	NT	640
2	Kano	2	F	63,022	10,998	4,585	320
3	Adamawa	6	M	63,146	41,200	20,627	1,280
4	Kano	2	M	63,213	63,331	63,353	1,280
5	Sokoto	2	F	63,123	8,215	–	80
6	Borno	7	M	63,173	13,873	7,471	160
7	Borno	6	F	63,065	63,065	NT	2,560
8	Sokoto	7	F	64,118	63,285	54,669	640
9	Borno	6	M	63,592	28,033	NT	80
10	Sokoto	6	F	64,176	63,427	35,190	640
11	Sokoto	2	F	–	NT	NT	<20
12	Adamawa	7	M	–	NT	NT	<20
13	Unknown	7	M	–	NT	NT	<20
14	Kano	7	M	–	NT	NT	<20
Ethiopia							
1	Somali	5	F	63,592	63,357	50,563	640
2	Afar	6	F	63,341	63,005	NT	2,560
3	Afar	13	F	63,366	63,205	63,467	1,280
4	Afar	10	F	63,206	63,299	NT	640
5	Afar	5	F	63,466	10,583	5,911	160
6	Fentale	<4	M	63,408	63,480	60,135	1,280
7	Afar	4	F	63,476	33,909	19,161	80
8	Afar	4	F	–	NT	NT	<20
9	Afar	2	M	–	NT	NT	<20
10	Afar	1	F	10,937	NT	NT	<20
11	Afar	3	F	18,269	NT	NT	<20
12	Fentale	>8	F	63,486	23,654	10,246	1,280
13	Afar	6	F	63,496	63,380	53,030	1,280
14	Afar	1	F	63,401	19,087	9,834	80
Tunisia							
1	Sidi Bouzid	8	F	–	NT	NT	<20
2	Sidi Bouzid	8	F	63,217	20,620	NT	80
3	Sidi Bouzid	6	F	–	NT	NT	<20
4	Sidi Bouzid	1	M	–	NT	NT	<20
5	Kebili	7	M	63,139	–	–	320
6	Kebili	4	M	63,113	–	–	160
7	Sidi Bouzid	1	M	–	NT	NT	<20
8	Sidi Bouzid	9	F	63,005	17,821	9,652	80
9	Sidi Bouzid	6	F	–	NT	NT	<20
10	Kebili	4	M	63,120	18,320	9,732	160
11	Sidi Bouzid	<1	M	–	NT	NT	<20
12	Sidi Bouzid	2	F	63,060	63,236	63,366	2,560
13	Sousse	13	F	63,220	50,510	26,575	320
14	Sidi Bouzid	5	F	–	NT	NT	<20

*MERS S1, Middle East respiratory syndrome coronavirus S1 micro-array. Serum dilutions tested 1:20, 1:320 or 1:640. Results expressed as relative mean fluorescent intensity (RFU) for each set of quadruplicate spots of antigen, with a cutoff of 4,000 RFU and >63.000 RFU as saturation signal; VNT, virus neutralization test (highest neutralizing serum dilution indicated); NT, not tested; –, negative.

## Conclusions

Since the discovery of MERS-CoV in 2012, accumulating serologic and molecular evidence demonstrates that the virus in dromedaries is genetically very similar to MERS-CoV in humans and points to the conclusion that dromedary camels are reservoirs for human infection. MERS-CoV genomic fragments have been detected in dromedaries in Qatar (*10*) and Saudi Arabia (*9*); near full-genome sequences have been generated from dromedaries in Egypt (*5*) and full-genome sequences have been generated from dromedaries in Saudi Arabia (*13*). Here, we show serologic evidence for circulation of MERS-CoV or MERS-like CoV in dromedaries in countries in East, West, and North Africa, with possible herd-specific differences in prevalence in Tunisia. The lower seropositivity observed in herds raised for meat production in Tunisia might reflect a high turnover of camels with a continuous introduction of animals unexposed to the MERS-CoV into these herds. No camels imported from neighboring countries were found at the meat-producing farms in Sidi Bouzid and Sousse, only camels purchased from other farms in the same area or other areas in Tunisia. However, animals are frequently moved between Libya and Kebili for trade.

**Figure 2:**
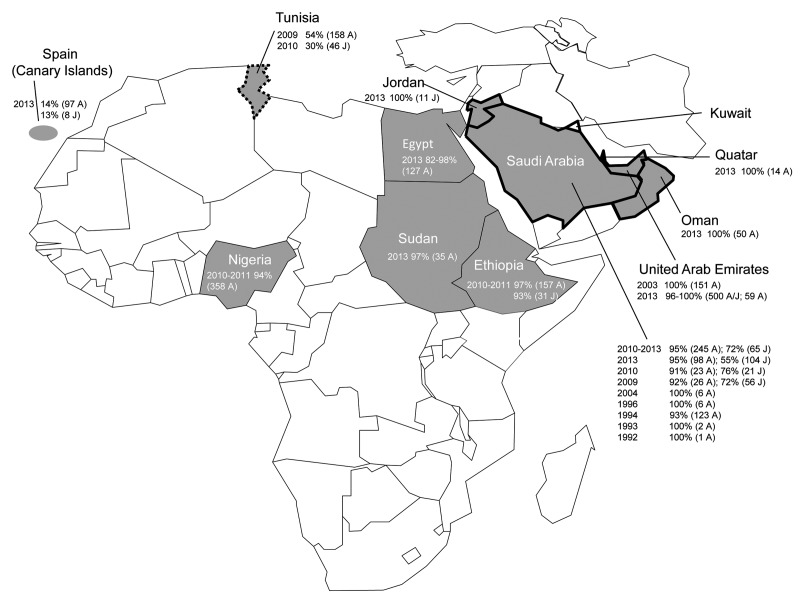
Geographic distribution of serologic evidence for Middle East respiratory syndrome coronavirus (MERS-CoV) or MERS-like CoV circulation in dromedaries in Africa and the Arabian Peninsula. Gray shading indicates countries with seropositive dromedaries; solid black outline indicates countries with primary human cases; dotted outline indicates countries with secondary human cases. For each country with affected dromedaries, the year of sampling, % seropositive, total number tested, and age group are indicated. A, adult, >2 years of age; J, juvenile, ≤2 years of age. Details on serologic tests used and references are in Table 1.

Samples in this study were collected during 2009–2011, confirming observations by us and others (*6*) that the virus circulated well before March 2012, which is the estimated time of identification of the most common ancestor for the MERS-CoV strains found in humans to date (*14*). The earliest serologic indication for circulation of MERS-CoV or MERS-like CoV in dromedaries was observed in 1992; however, this result was based on results of a whole-virus ELISA with undescribed specificity (*9*). On the basis of well-validated array and neutralization tests, the study of dromedaries in the United Arab Emirates showed the presence of MERS-CoV or MERS-Cov-like antibodies as early as 2003 (*6*). The accumulated data on MERS-CoV serology in dromedaries (Figure 2; Table 1) show circulation of MERS-CoV or MERS-like CoV in dromedaries in Africa and the Arabian Peninsula well before 2012, when the first cases in humans were identified, and show overall high levels of seropositivity, including in animals from countries without reported human cases. 

A question raised by these findings is whether human cases occur outside the Arabian Peninsula and if such cases are currently underdiagnosed in Africa. In addition, for the whole region, the possibility exists that MERS-CoV illness occurred before its discovery in 2012 and that such infection has been overlooked in the areas with evidence for virus circulation among animals during the past 10 years. Retrospective studies of cohorts of humans with respiratory illnesses of unknown etiology should address this notion. 

Alternative explanations for the lack of cases in Africa could be the following: a different risk profile, for instance, related to demographics and local practices; or subtle genetic differences in the circulating virus strain. Full-genome sequencing, virus isolation, and phenotypic characterization of viruses circulating outside the Arabian Peninsula will resolve this issue. Meanwhile, awareness of MERS-CoV infections should be raised among clinicians in Africa.
